# Micronutrient supplements as antioxidants in improving sperm quality and reducing DNA fragmentation

**DOI:** 10.1186/s12610-023-00197-9

**Published:** 2023-09-14

**Authors:** Nguyen Dac Nguyen, Minh Tam Le, Nhu Quynh Thi Tran, Quoc Huy Vu Nguyen, Thanh Ngoc Cao

**Affiliations:** 1 Center for Reproductive Endocrinology and Infertility, Hue University of Medicine and Pharmacy, Hue University, 06 Ngo Quyen Street, Hue, 53000 Vietnam; 2 Department of Obstetrics and Gynecology, Hue University of Medicine and Pharmacy, Hue University, Hue, Vietnam

**Keywords:** Male infertility, Sperm DNA fragmentation, Antioxidants, Oxidative stress, Reactive oxygen species, Infertilité masculine, Fragmentation de l'ADN des s*Spermatozoïdes, Antioxydants, Stress oxydatif, Espèces réactives de l'Oxygène

## Abstract

**Background:**

Spermatogenesis and sperm quality may be negatively impacted by an increase in reactive oxygen species. This study investigates the efficacy of combined antioxidant therapy for treating male infertility, as measured by semen analyses and the sperm DNA fragmentation index (DFI). Infertile men with a high sperm DNA fragmentation index were instructed to take two oral micronutrient capsules daily for three months. Each antioxidant formulation contained 60 mg vitamin E, 400 µg folic acid, 30 mg selenium, 125 mg L-arginine, 220 mg L-carnitine, 7.5 mg coenzyme Q10, 40 mg L-glutathione, and 20 mg zinc citrate. At entry and post-treatment, the general characteristics, semen analysis, and sperm chromatin dispersion assays were recorded and compared.

**Results:**

After three months of treatment with antioxidant compounds, the quality of spermatozoa improved significantly, as indicated by a decrease in the mean DNA fragmentation index from 45.6 ± 17.2% to 34.8 ± 20.3%; an increase in sperm concentration from 29.7 × 10^6^/mL to 35.7 × 10^6^/mL (*p* < 0.001), an increase in a total number of spermatozoa from 72.1 × 10^6^ to 95.5 × 10^6^ (*p* = 0.012), and an increase in the vitality from 75.5 ± 17.1 to 81.1 ± 14.4% viable forms (*p* < 0.001).

**Conclusions:**

Micronutrient supplementation can improve sperm quality and DNA integrity in infertile men. Men with infertility and significant sperm DNA fragmentation who take antioxidants for three months experience a reduction in DNA fragmentation index and an increase in sperm quality as measured by the semen analysis.

**Trial registration:**

NCT04509583. Registered 12 August 2020, Hue University of Medicine and Pharmacy Ethics Committee—Retrospectively registered.

## Résumé

**Contexte:**

La spermatogenèse et la qualité du sperme peuvent être affectées négativement par une augmentation des espèces réactives de l'oxygène. La présente étude évalue l'efficacité d’une thérapie antioxydante combinée pour traiter l'infertilité masculine, telle que mesurée par les analyses du sperme et l'indice de fragmentation de l'ADN des spermatozoïdes (DFI). Les hommes infertiles avec un indice de fragmentation de l'ADN des spermatozoïdes élevé ont été invités à prendre, par voie orale, deux capsules de micronutriments par jour pendant trois mois. Chaque formulation antioxydante contenait 60 mg de vitamine E, 400 μg d'acide folique, 30 mg de sélénium, 125 mg de L-arginine, 220 mg de L-carnitine, 7,5 mg de coenzyme Q10, 40 mg de L-glutathion et 20 mg de citrate de zinc. À l'entrée et après le traitement, les caractéristiques générales, l'analyse du sperme et les tests de dispersion de la chromatine spermatique ont été enregistrés et comparés.

**Résultats:**

Après trois mois de traitement avec des composés antioxydants, la qualité des spermatozoïdes s'est considérablement améliorée, comme l'indique une diminution de l'indice moyen de fragmentation de l'ADN de 45,6±17,2% à 34,8±20,3%; une augmentation de la concentration de spermatozoïdes de 29,7×106/mL à 35,7×106/mL (*p*<0,001), une augmentation du nombre total de spermatozoïdes de 72,1x106 à 95,5x106 (*p*=0,012), et une augmentation de la vitalité de 75,5±17,1 à 81,1±14,4% des formes viables (*p*<0,001).

**Conclusions:**

La supplémentation en micronutriments peut améliorer la qualité du sperme et l'intégrité de l'ADN chez les hommes infertiles. Les hommes souffrant d'infertilité et d'une fragmentation importante de l'ADN des spermatozoïdes qui prennent des antioxydants pendant trois mois subissent une réduction de l'indice de fragmentation de l'ADN et une augmentation de la qualité du sperme, mesurée par l'analyse du sperme.

## Introduction

Male factors account for around fifty percent of infertility cases [1]. Several causes of male infertility, such as endocrine disorders, varicocele, vas deferens obstruction, genital infection or ejaculatory failure, and sexual dysfunction, have been found; however, more than 30% of cases are idiopathic [2]. Analysis of sperm parameters has been the conventional method for assessing male fertility. In recent decades, sperm DNA fragmentation (SDF) has been utilized as a highly accurate predictor of sperm quality and function [3]. Higher levels of SDF are related to a longer time to conceive, poorer pregnancy outcomes throughout treatment cycles, and an increased risk of miscarriage [4]. SDF is crucial in defining the reproductive capability of males.

Multiple factors, including radiation, medication, tobacco and alcohol usage, diet, the environment, varicocele, and oxidative stress (OS), have been implicated in the increase in SDF. OS is an imbalance between reactive oxygen species (ROS) and antioxidant quantities [5]. ROS are composed of oxygen radicals and non-radical derivatives. Among them, oxygen radicals are synthesized by spermatozoa through metabolic activities, sperm mitochondrial activity, or activity of leukocytes, and are crucial for cell signal transmission, sperm maturation, and sperm acrosome responses [6]. However, increasing levels of ROS may have detrimental effects on spermatogenesis and sperm quality. Numerous investigations have demonstrated that an increase in ROS capacity damages spermatozoa's structure and physiological function of spermatozoa [7]. Exogenous factors (environmental, exposure to risk factors, testicular hyperthermia) and some endogenous factors (immature spermatozoa, leucocytes, and varicocele) have been identified as causes of high ROS levels. In addition, when triggered by an infection, neutrophils can produce excessive quantities of ROS, resulting in oxidative damage to sperm DNA [8]. Various SDF assays and OS measurements have improved male fertility potential observation [9].

For optimal sperm function, it is essential to maintain a balance between reactive oxygen species and antioxidants. Antioxidants are present in the seminal fluid predominantly in two forms: non-enzymatic (vitamins D, E, C, and B, Coenzyme Q10, pyruvate, glutathione, carnitines, and trace metals) and enzymatic (catalase, superoxide dismutase, glutathione peroxidase) [10]. Consequently, analyzing and lowering OS with antioxidant therapy may be a viable infertility care strategy. Previous research supports using antioxidant supplements to decrease OS levels and improve pregnancy outcomes [11, 12]. However, there is no high level of agreement among studies on treatment procedures (dosage, combinations of compounds, and outcome measures).

Antioxidant preparations have been demonstrated to enhance reproductive and sperm functions. First, vitamin E, selenium, and glutathione counteract the ROS levels in seminal fluid [13]. Second, zinc, folic acid, and selenium promote DNA synthesis and the protamine packaging of sperm chromatin [14]. Finally, carnitine, arginine, and coenzyme Q10 are crucial in transporting fatty acids into mitochondria for energy production [15]. This study examined the efficacy of a combination antioxidant therapy in treating male infertility, employing semen analysis and SDF as outcome measures.

## Methods

### Study design

This interventional trial was conducted at a tertiary university hospital between November 2019 and March 2021. Men from infertile couples, as defined by World Health Organization standards [16], who had a high DNA fragmentation index (DFI ≥ 30%) [17] met the inclusion criteria. Men with azoospermia, retrograde ejaculation, infection, acute systemic disorders, malignant diseases, hepatic function problems, or using antioxidant compounds or vitamins during the past two months were excluded from the study.

The following patient characteristics were recorded: age, occupation, history of measles, chronic conditions, smoking, drinking habits, and physical examination. Anthropometry, biochemical tests, semen analyses, and sperm chromatin dispersion (SCD) assays (the Halosperm test) were performed for all participants.

In this study, the sample size was determined from the formula N = 2C(1 – r)/ES2 with the following parameters: ES: Standardized difference (ES =  × 1 −  × 0/s0; where × 1 and × 0 are the value of DFI before and after treatment with antioxidants and s0 is standard deviation: × 1 = 43.5%, × 0 = 34.3%, s0 = 22.8; ES = 0.40 [18]), r: Correlation Coefficient; C: acceptable difference (power = 0.95, and α = 0.05, C = 13,000). The expected size of the sample was 32 cases. Seventy-one men were recruited for the current investigation.

All procedures were executed in compliance with the applicable guidelines. All participants provided informed consent in accordance with the Helsinki Declaration of 2013.

### Anthropometry

The body mass index (BMI)_of each participant was calculated by dividing their weight (in kilograms) by the square of their height (in meters). The hip circumference was measured at the level of the pubic symphysis. At the end of expiration, the waist circumference (WC) was measured at the umbilicus level.

### Biochemical assays

Levels of low-density lipoprotein cholesterol (LDL-C), high-density lipoprotein cholesterol (HDL-C), triglycerides, total cholesterol, fasting glucose levels, and oral glucose tolerance test results were measured with a Roche/Hitachi Cobas system (Module COBAS 4000/6000, Roche Diagnostics, Indianapolis, IN, USA). In the morning, the blood sample was collected after an overnight fast. Tubes with anticoagulant plasma, Li-heparin, and K2-EDTA serum were used for taking and preparing specimens. Samples in the tubes were handled according to the tube manufacturer’s instructions.

### Semen analysis

The WHO 2010 guidelines were followed to evaluate the sperm analysis results [19]. Masturbation samples of semen were taken after 3 − 5 days of ejaculatory abstinence. The sample was collected into a sterile, wide-mouthed container. After being liquefied, the samples were analyzed within an hour of their collection. Time of liquefaction, pH, volume, total sperm count, motility, concentration, morphology, vitality, and leukocyte count were assessed. Sperm vitality was determined by seeing sperm stained with eosin Y and observed under a 40X microscope. The sperm morphology was assessed by evaluating the head's size and shape and the features of the midsection and tail.

### Sperm chromatin dispersion assay

**Fig. 1:**
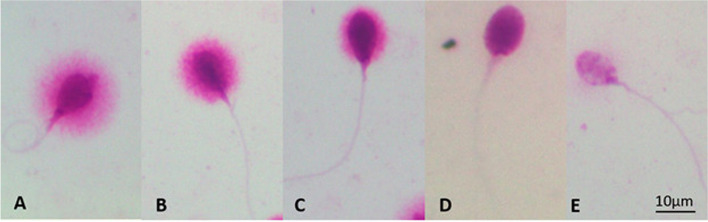
Images of sperm chromatin dispersion test are assessed from halo types: normal group includes (**A**) big halo spermatozoa (halo thickness equal to or larger than the length of the minor diameter of the core) and (**B**) medium halo spermatozoa (thickness less than the length of the minor diameter of the core and larger than 1/3 of the minor diameter of the core); abnormal group includes (**C**) small halo spermatozoa (thickness equal to or less than 1/3 diameter of the minor diameter of the core), (**D**) without halo spermatozoa, and (**E**) degraded spermatozoa (sperm show no halo and present a core irregularly or weakly stained, represent a subpopulation of spermatozoa with extensive DNA and nuclear protein damage)

SDF was assessed utilizing the Halosperm kit from Halotech DNA, SL (Spain). The sperm samples were diluted with Phosphate Bufferd saline to a 5 − 10 million sperm per milliliter, and 25 µL of material was combined with Eppendorf agarose; the cell suspension was applied to the treated side (available from Halotech DNA’s Halo sperm kit) of the microscope slide which was then refrigerated for 5 min at 4^0^C. After taking the slide out of the fridge, immediately immerse the slide into the denaturant agent solution to denature DNA in cells with fragmented DNA, which contained 10 µL of distilled water and 80 µL of HCl; it was incubated for 7 min at room temperature. The slide was then incubated for 25 min at room temperature in 10 mL lysis solution before being washed with distilled water for 5 min. Afterward, it was placed in ethanol (70% for 2 min, 90% for 2 min, and 100% for 2 min). Slides were monitored for SDF in a fluorescence microscope following their drying. Following Fernandez's criteria [17], we screened 500 spermatozoa and classified each spermatozoon as having fragmented DNA as shown in Fig. 1. Following the observation, the total score for each halo type was determined. DFI was determined by dividing the number of spermatozoa with fragmented DNA by the total number of cells evaluated. A DFI result of less than 30% was considered normal [20].

### Intervention with micronutrient supplements

Men with a DFI equal to or above 30% were instructed to consume antioxidant preparations daily for three months. Physiologically, as spermatogenesis and spermiogenesis take an average of three months, the minimum time required to detect a change in sperm quality is also three months. In addition, we refrained from extending the length of therapy for too long out of worry that a continuous usage of antioxidants would induce a redox reaction, which could have negative consequences on sperm.

Each antioxidant preparation contained 60 mg vitamin E (DL-alpha-tocopheryl acetate), 400 µg folic acid, 30 mg selenium, 125 mg L-arginine, 220 mg L-carnitine, 7.5 mg coenzyme Q10, 40 mg L-glutathione, and 20 mg zinc citrate. The above compounds were used in capsule form (Profortil, Abbott, US). The patient was prescribed two tablets a day for three months. After the treatment period, all individuals underwent a medical examination, metabolic testing, semen analysis, and SCD tests to determine the treatment's efficacy.

### Statistical analysis

Descriptive statistics established the characteristics of the study population. The Kolmogorov–Smirnov test was used to test the normal distribution of the research variables. The assessment for equality of variances was effectuated by Levene's Test. A Paired—Sample T-test (normally distributed variable) or Wilcoxon matched-paired signed rank (non-normally distributed variable) was employed on independent samples to examine changes between variables before and after therapy. Pearson correlation test was performed to test the relation between two research variables. Results were presented as mean (95% confidence interval) or %. Indicative of statistical significance was a *p*-value less than 0.05. SPSS version 20.0 (IBM Co., Armonk, NY, US) was used for data analysis. Cases without follow-up data were excluded.

## Results

Table 1 presents the anthropometric statistics of men with infertility. The majority of the patients fell under the category of primary infertility. Most males were infertile for at least three years and had no history of mumps.

**Table 1:** Baseline characteristics of male in infertile couples

**Characteristics**	**Number**	**(%)**
**Age (years)**		
Mean: 35.31 ± 6.08 (33.87—36.75)		
< 35	31	43.7
≥ 35	40	56.3
**Infertility types**		
Primary	44	62.0
Secondary	27	38.0
**Infertility duration (years)**		
Mean: 4.45 ± 2.52 (3.85—5.05)		
< 3	21	29.6
≥ 3	50	70.4
**History of mumps**		
Yes	7	9.9
No	64	90.1
**Alcohol consumption**		
Yes	39	54.9
No	32	45.1
**Triglycerides (mmol/L)**	2.0 ± 1.2	
**Total cholesterol (mmol/L)**	4.7 ± 0.8	
**HDL-C (mmol/L)**	1.3 ± 0.4	
**LDL-C (mmol/L)**	3.1 ± 0.8	
**Glucose (mmol/L)**	5.5 ± 1.4	

Data presented as mean ± standard deviation or number (percentage)

*HDL-C* High density lipoprotein, *LDL-C* Low density lipoprotein

As shown in Table 2, after three months of antioxidant therapy, the average SDF index decreased significantly that indicates a considerable improvement in sperm quality. The rate of deteriorated spermatozoa and those without a halo significantly reduced over the course of three months. The population of spermatozoa with big halos increased significantly with *p* = 0.049. Other results, including populations of spermatozoa with small and medium halos, exhibited no significant change (*p* > 0.05).

**Table 2:** The results of semen characteristics before and after treatment

Semen characteristics	Before treatment n (%)	After treatment n (%)	*p* value^1^
**Semen analysis**			
Abnormal	60 (84.5)	52 (73.2)	0.100
Normal	11 (15.5)	19 (26.8)	
**Total number (10**^**6**^**)**	**72.1 (58.2 – 85.3)**	**95.5 (76.8 – 112.0)**	0.012**
**Volume (mL)**	**2.4 (2.14 – 2.62)**	**2.6 (2.38 – 2.89)**	0.133**
< 1.5	13 (18.3)	5 (7.0)	0.044
≥ 1.5	58 (81.7)	66 (93.0)	
**Concentration (10**^**6**^**/mL)**	**29.7 (25.8 – 33.5)**	**35.7 (30.9 – 39.8)**	< 0.001*
< 15	11 (15.5)	8 (11.3)	0.460
≥ 15	60 (84.5)	63 (88.7)	
**Motility (%)**	**27.5 (24.4 – 30.2)**	**27.7 (24.9 – 30.4)**	0.990*
< 32	24 (33.8)	29 (40.8)	0.386
≥ 32	47 (66.2)	42 (59.2)	
**Vitality (%)**	**75.5 (71.1 – 79.5)**	**81.1 (77.5 – 84.6)**	< 0.001**
< 58	5 (7.0)	3 (4.2)	0.467
≥ 58	66 (93.0)	68 (95.8)	
**Normal morphology (%)**	**3.2 (2.7 – 3.7)**	**3.4 (3.0 – 3.8)**	0.089**
< 4	47 (66.2)	37 (52.1)	0.088
≥ 4	24 (33.8)	34 (47.9)	
**Sperm DNA fragmentation**			
Big halo	81.8 (63.5 – 99.4)	118.1 (93.4 – 145.5)	0.049**
Medium halo	190.1 (168.8 – 210.3)	205.3 (181.9 – 223.4)	0.320*
Small halo	93.3 (80.4 – 107.4)	81.9 (71.5 – 93.5)	0.449**
Without halo	86.7 (73.2 – 101.6)	59.4 (46.4 – 73.2)	0.002**
Degraded sperm	48.4 (38.2 – 57.4)	30.3 (24.9 – 35.7)	< 0.001**
DFI %	45.6 (41.6 – 50.0)	34.8 (30.0 – 40.0)	< 0.001**

^1^Data are presented as mean (95% Confidence interval) or number (%)

^*^Paired—Sample T test (normally distributed variable)

^**^Wilcoxon matched—paired signed rank test (non—normally distributed variable)

The sperm analysis revealed an increase in concentration and viability. After three months of treatment, other fundamental semen characteristics (semen volume, total sperm count, sperm motility, and morphology) were not altered significantly. Semen analysis was considered abnormal if at least one parameter was not in the WHO reference range. The percentage of patients with normal sperm analysis increased clearly after therapy, but this was not statistically significant (Table 2).

As shown in Table 3, antioxidant supplementation did not affect anthropometric features or other relevant parameters (WC, hip circumference, weight, BMI, waist-to-height ratio, waist-to-hip ratio; *p* > 0.05). Although correlations between semen parameter values and DFI were not found in pre-treatment patients (*p* > 0.05), there was a negative association between the level of DFI and the percentages of motile and vital spermatozoa three months post-treatment, as shown in Table 4. A positive association was also identified between DFI and the proportion of spermatozoa with abnormal necks and tails.

**Table 3:** The changes in other relevant factors after treatment

**Factors**	**Before treatment** **n (%)**	**After treatment** **n (%)**	***p* value**^**1**^
**Alcohol**			
Yes	39 (54.9)	38 (53.5)	0.866
No	32 (45.1)	33 (46.5)	
**Smoking**			
Yes	18 (25.4)	15 (21.1)	0.551
No	53 (74.6)	56 (78.9)	
**Waist (cm)**	84.8 (82.9 – 86.6)	84.3 (82.5 – 86.1)	0.225*
**Hips (cm)**	96.2 (94.8 – 97.6)	95.9 (94.7 – 97.2)	0.257*
**Weight (kg)**	64.6 (62.8 – 66.6)	64.7 (62.9 – 66.7)	0.133**
**BMI (kg/m**^**2**^**)**	23.0 (22.4 – 23.6)	23.1 (22.4 – 23.7)	0.124*
**WHR (%)**	0.9 (0.8 – 0.9)	0.9 (0.8 – 0.9)	0.553*
**WHtR (%)**	0.5 (0.4 – 0.5)	0.5 (0.5 – 0.5)	0.221*

*BMI* Body mass index, *WHR* waist- hip ratio, *WHtR* waist-to-height ratio

^1^Data are presented as mean (95% Confidence interval) or number (%)

^*^Paired—Sample T test (normally distributed variable)

^**^Wilcoxon matched—paired signed rank test (non—normally distributed variable)

**Table 4:** Correlations between semen parameters and sperm DNA fragmentation index in participants before and after treatment

**Sperm factors**	**% DFI before treatment**		**% DFI after treatment**	
	**r**	*p* value^*^	**r**	*p* value^*^
**Concentration**	0.037	0.758	-0.207	0.083
**Motility**	-0.021	0.860	-0.431	< 0.001
**Vitality**	0.019	0.877	-0.486	< 0.001
**Normal morphology**	-0.004	0.975	-0.229	0.055
**Abnormal head**	-0.009	0.940	0.012	0.920
**Abnormal neck and tail**	-0.044	0.719	0.299	0.011

*DFI* DNA fragmentation index

^*^Pearson correlation test

## Discussion

It has been reported that oxidative stress has a deleterious effect on male fertility, resulting in aberrant semen parameter values and increased SDF levels. Spermatozoa cells are more susceptible to oxidative stress than other cells because they contain less cytoplasm and more unsaturated fatty acids. The ROS and antioxidant levels imbalance may result in DNA and sperm plasma membrane peroxidation [21]. Therefore, it is anticipated that the quality of spermatozoa or male fertility will depend on the availability of antioxidants in seminal plasma. In this study, sperm concentration and vitality increased significantly after therapy. Several previous articles have found inconsistent conclusions about the efficacy of antioxidant supplementation in men with infertility, but the majority have suggested positive effects. In an observational trial involving 690 individuals with astheno-teratozoospermia who received 400 mg vitamin E and 200 selenium daily for 100 days, 52.6% of males experienced a significant increase in the proportion of motile spermatozoa, normal sperm morphology, or both (*p* < 0.001) [22]. Comhaire et al. conducted a prospective trial on 27 men with infertility and concluded that the combination of vitamins A and E and essential fatty acids increased sperm concentration in oligozoospermic individuals [23]. Compared with the placebo group, all semen parameter values improved in participants with varicocele and idiopathic infertility treated with L-carnitine [24]. In another double-blind interventional research with 211 subfertile men randomly assigned to four groups (zinc only, folic acid only, folic acid and zinc, and placebo), a significant rise in normal sperm concentration was reported in the group receiving combined therapy [25]. Using coenzyme Q10 as an antioxidant supplement for three months may improve semen parameter values (sperm concentration, progressive motility, and total motility), oxidative stress indicators, and SDF in men with infertility, particularly those with idiopathic oligoasthenozoospermia [26]. In a recent Cochrane meta-analysis involving men with abnormal sperm analysis results, the combination of vitamin E, selenium, and N-acetylcysteine for three months increased the incidence of motile sperm by 12% compared with placebo [11].

On the contrary, some authors have failed to find any significant effects of micronutrient supplementation in infertile men. Rolf et al. did not observe the positive effects of taking vitamin E (800 mg) and vitamin C (1000 mg) daily for 56 days on semen quality [27]. Treatment with 1000 mg L-carnitine and 500 mg L-acetyl-carnitine daily for three months did not improve the results [28]. Menevit (25 mg zinc + 100 mg vitamin C + 400 IU vitamin E + 6 mg Lycopene + 333 µg garlic oil) daily for three months did not improve routine sperm parameters in men with high SDF tested by terminal deoxynucleotidyl transferase dUTP nick end labeling (TUNEL) [13]. Another recent MOXI (In the Males, Antioxidants, and Infertility) study used the following antioxidant formulation: 1000 µg folic acid + 10 mg lycopene + 0.2 mg selenium + 500 mg vitamin C + 400 mg vitamin E + 20 mg zinc + 1000 mg L-carnitine + 2000 IU vitamin D, had no beneficial effect on semen parameters [29]. Published studies do not reach a consensus on the treatment regimen or duration. An overdosage of antioxidant supplements may be an adverse factor affecting semen parameters. Furthermore, the balance between ROS and antioxidant systems is crucial to obtain optimal sperm function; the overconsumption of antioxidants may generate reductive stress that could impair mitochondrial activity and negatively affect human reproductive health [30]. The results of our study with a combination of specific micronutrients have confirmed the effectiveness of improved sperm parameters.

Several authors have identified a correlation between SDF and semen parameter values. Similarly, we found a negative correlation between DFI results and the extent of sperm motility and vitality. A positive correlation between DFI and the percentage of spermatozoa with abnormal necks and tails was also observed. Moreover, the mean SDF level decreased and the rate of degraded spermatozoa and those without halo also showed a sharp decrease after three months of treatment. Logically, the decrease in DFI levels is expected to improve sperm quality, although the percentage of motile spermatozoa was not reduced by using micronutrient supplements. Sperm DNA may be more sensitive to antioxidants than the mitochondria or sperm membrane (determinants of motility) [13]. Therefore, if ROS is partially neutralized by antioxidant supplementation, the remaining ROS might no longer be sufficient to attack the DNA; however, they may still inhibit mobility.

Regarding the benefits of oral antioxidants supplement in sperm DNA integrity, Arafa et al. have indicated ‘FH PRO for men’ (Multivitamin support for male fertility contains: L-Carnitine, Arginine, Zinc, CoQ10, Lycopene; Fairhaven Health, US) on 148 men with infertility and realizes that those with idiopathic infertility had improvement in semen parameters, oxidation–reduction potential, and SDF evaluated by the Halosperm kit [18]. Coenzyme Q_10_ supplementation for three months has also been shown to reduce ROS levels and SDF levels in patients with oligoasthenozoospermia [26]. In another interventional controlled study on 64 patients with DFI > 15%, SDF was determined by the TUNEL method [31]; after two months of taking vitamins C and E, DFI significantly decreased from 22 to 9%. However, a recent study in 2020 did not determine the positive effects of antioxidants on DNA integrity [29], and the difference in the decrease in DFI tested by Sperm Chromatin Structure Assay (SCSA) was not statistically significant between the antioxidant group and placebo (*p* = 0.548). Steiner et al. suggested that the difference in the antioxidant formulation and inclusion criteria in previous studies were the main reason. The need for an optimal antioxidant formulation is very important either to reduce the potential side effects of reductive stress, antioxidants or improve the quality of spermatozoa effectively. Identifying the target patient group that is likely to benefit the most from the use of antioxidants is also an urgent problem.

### Limitations of the study

Logically, men with seminal oxidative stress are presumably the most likely to benefit from antioxidant therapy. In our investigation, oxidative stress in semen samples was not evaluated. If these data included information on ROS levels in relation to DFI and therapeutic efficacy in accordance with ROS levels, our inquiry will be more persuasive. In addition, if there is evidence of a lack of specific nutrients and antioxidant radicals in sperm, our investigation will be more beneficial. In reality, we did not incorporate this information in our analysis. To confirm the precise benefits of this regimen, there is an urgent need for trials evaluating the efficiency of antioxidant treatment in persons with semen oxidative stress.

## Conclusion

Micronutrient supplementation can improve semen parameter values and DNA integrity in men with infertility. The study shows that using antioxidants for a duration of three months decreases SDF and elevates the results of routine semen analyses in men with infertility with high SDF.

## Data Availability

The dataset used and/or analyzed during the current study is available from the corresponding author upon reasonable request.

## Abbreviations

SDF: Sperm DNA fragmentation
OS: Oxidative stress
ROS: Reactive oxygen species
WHO: World Health Organization
DFI: DNA fragmention index
SCD: Sperm chromatin dispersion
ES: Standardized difference
BMI: Body mass index
WC: Waist circumference
LDL-C: Low-density lipoprotein cholesterol
HDL-C: High-density lipoprotein cholesterol
TUNEL: Terminal deoxynucleotidyl transferase dUTP nick end labeling
SCSA: Sperm Chromatin Structure Assay
